# Fighting against HIV and AIDS within a resource constrained rural setting: a case study of the *Ruvheneko* Programme in Chirumhanzu, Zimbabwe

**DOI:** 10.1080/17290376.2019.1605537

**Published:** 2019-04-17

**Authors:** Christina Tafadzwa Dzimiri, Patrick Dzimiri, Kezia Batisai

**Affiliations:** a Department of Sociology, University of Johannesburg, Johannesburg, South Africa; b Department of Development Studies, University of Venda, Thohoyandou, South Africa

**Keywords:** HIV/AIDS, rural initiatives, Ruvheneko, Community Home-Based Care, community engagement

## Abstract

Since reports of the first incidence of the HIV virus in Zimbabwe in 1985, the epidemic has negatively impacted on every facet of human security. Rural areas, by virtue of being the periphery and constrained in terms of resources and health care provision, bear the brunt of the epidemic. In light of the above background, this paper examined how the establishment of *Ruvheneko* Programme by the people of Chirumhanzu helped in mitigating on the impact of HIV and AIDS in the rural sphere. The paper analyses how the community of Chirumhanzu successfully engaged each other to the extent of coming up with such a vibrant programme. This is raised against the backdrop of failure usually associated with HIV and AIDS engagement projects. The study made use of field interviews and focus group discussions as data collection instruments. Participants were purposively selected on the basis of their knowledge and participation in the establishment and activities of Ruvheneko Programme. Selected were 5 St Theresa’s Hospital Staff, 10 Roman Catholic Church members of which, 5 were from the St Anna’s woman prayer group and 5 men from St Joseph’s men prayer group, 1 village head and 2 elders from the same nearby village constituted key informants. Complementing the use of interviews and focus group discussions was the analysis of secondary data sources on HIV and AIDS in Zimbabwe as well as the Ruvheneko Programme. To understand the collective role of various sectors of the community in establishing Ruvheneko Programme, the paper derives insights from the perspective of social capital theory and its notion of commonality to strengthen communities. Findings from the study show that, unlike other HIV and AIDS programmes that are exported from the urban to the rural areas, *Ruvheneko* Programme demonstrates a grassroots-level response to HIV and AIDS. Again, social cohesion fostered by aspects such as religiosity, cultural ethos of *Ubuntu*, and a consultative approach played a key role in unifying people towards fighting HIV and AIDS in Rural Chirumhanzu.

## 1. Introduction and background

The first report on HIV and AIDS in Zimbabwe was in 1985 where an estimated 119 cases of HIV infections were recorded countrywide (Broom & O’Brien, 2011; Mapenzauswa, 2004; Rembe, 2006; United Nations Programme on HIV and AIDS [UNAIDS], 2014; Zimbabwe National HIV and AIDS Strategic Plan (ZNASP), 2006–2010). Since then, the HIV and AIDS epidemic spread rapidly throughout the country and the mode of transmission has been mainly heterosexual contact which constitutes 92%, while perinatal transmission accounts for 7% and others 1% (Rembe, 2006; ZNASP, 2006–2010). A study conducted in 2003 by the United Nations International Children’s Emergency Fund (UNICEF) reveals that 1.3 million children were orphaned due to HIV and AIDS related cases, and 30% of these orphans were from rural and high density areas (UNICEF, 2003). By the year 2005, national infection estimates were at 1.6 million out of a population of 11.6 million. In addition, 55% of those known to be infected were women between 15 and 49 years, while those between 15 and 29 years were rated as the most vulnerable (ZNASP, 2006–2010). The youth are the most endangered group, with 18% of the youth between 15 and 24 years of age believed to be HIV positive (ZNASP, 2006–2010). Mother-to-child transmission (MTCT) of HIV has been the major cause of child mortality in Zimbabwe (Marinda et al., 2007), and is the most significant (90%) source of HIV infection in children under the age of 15 years (Duri, Stray-Pedersen, & Muller, 2013). Studies on the human security ramifications of HIV and AIDS demonstrated that the epidemic increases stress upon seriously affected families, societies, and may undermine the state’s capacity to provide the security needs of the country (Prince-Smith, 2007).

A 2009 report by the Physician for Human Rights laments that the government of Zimbabwe has failed to deliver on its responsibility to protect its people from all human security threats, including HIV and AIDS (Physicians for Human Rights, 2009). This was raised in the context of the deterioration of the health delivery system as a result of poor governance in the country (Batisai, 2013; MSF Report, 2009). The healthcare crisis was further aggravated by the exodus of health professionals in the period 2000–2009 (Physicians for Human Rights, 2009). Due to economic and political instability in the country, many professionals including those in the health sector had to explore green pastures beyond the frontiers of the country (Duri et al., 2013; Physicians for Human Rights, 2009). Other traumatic developments due to health care crisis include infant mortality rate which rose from 50 per 1000 live births in 1990 to 57 per 1000 live births in 2010 (UNAIDS (2014); World Health Organisation [WHO], 2006). Related developments include increase in adult mortality rate which rose from 286 per 1000 in 1990 to 751 per 1000 in 2006 (WHO, 2006). All these were amplified by diminished access to health care, the ‘brain drain’, closure of public hospitals, as well as prohibitive care costs exacerbated by shortage of foreign currency (Batisai, 2013, 2016; Duri et al., 2013).

The government response, which came very late in the 1990s, saw the introduction of the National AIDS Fund (also called AIDS Levy), where 3% of taxable incomes on individuals and corporations were to be channelled towards raising AIDS funds (National AIDS Council [NAC], 1999). Other mitigation measures that followed the introduction of the AIDS Levy included the integration of family planning with HIV, sexually transmitted infections (STI), and maternal health services; voluntary counselling and testing (VCT); and prevention of mother-to-child transmission (PMTCT) (Duri et al., 2013). That notwithstanding, a major shortcoming of these intercessions is that they did not prioritise the health needs of the rural population like Chirumhanzu. This population in Zimbabwe, similar to other rural folks on the continent, often encounters a myriad of challenges when trying to access healthcare centres (Batisai, 2016; Bene & Darkoh, 2014; Kautzky & Tollman, 2008; O’Brien & Broom, 2014).

Another significant response strategy by the government was the introduction of the Home-Based Care Policy in 2004 which accelerated the process of behaviour change. Home-Based Care envisages the process of transferring the duty of caring for the sick from the hospitals to the people’s homes so that they can be taken care of by their family and community members (Leff, 2017; Munshi & Bezuidenhout, 2017; NAC, 2010; Naylor & Keating, 2008). It is under the same context that Ruvheneko was established, hence the importance of discussing the establishment of Ruvheneko Programme.

### 1.1. Origins and establishment of Ruvheneko Programme

Ruvheneko Programme at St Theresa’s Hospital is situated in Chirumhanzu rural area in Midlands province of Zimbabwe and was established in 2003 as a rural initiative in mitigating the impact of HIV and AIDS in the area. HIV and AIDS had claimed many rural lives in Zimbabwe (Duffy, 2005; Duri et al., 2013; World Health Organisation [WHO], 2005), Chirumhanzu included. This was exacerbated by lack of knowledge of the disease and expertise on how to care for the sick. As alluded to previously, most of the government-led programmes on HIV and AIDS did not cater for the needs of the rural people. Coping strategies in Chirumhanzu in the early stages involved home care visits by the nurses, but they became overwhelmed and could not cope with their daily hospital duties as well as home visits. Upon realising this challenge which faced the hospital personnel, *Madzimai Enzanga* (women of the St. Anne’s Sect of the Roman Catholic Church (RCC) intervened by taking charge of the home visits. *Madzimai Enzanga* are also renowned for their involvement in helping the poor and sick with prayers, food stuffs, and clothes, even before the HIV and AIDS era. They started providing palliative and spiritual care to the infected and affected by helping in such tasks as bathing the sick, doing laundry, praying for the sick, watering gardens, weeding in the sick person’s agricultural fields as well as providing subsistence food stuffs. However, the increase in the number of HIV and AIDS related deaths, with an average of 15 people dying every month from AIDS related illnesses as well as high records of an average of 140 HIV and AIDS related cases per day, compelled the community, RCC, and the hospital authorities to join hands in the fight against HIV and AIDS (St. Theresa’s Hospital Report 1996–2002). They established what came to be known as the St Theresa’s HBC project in 1995 and this marked the origin of Community Home-Based Care (CHBC), though informal at this stage. An interest to pursue research on the Ruvheneko Programme was triggered by the publicity it received as a model for HIV and AIDS programme in a resource constrained rural setting (Jackson & Anderson, 2001). In its 2003 report, the Centre for Disease Control (CDC) hailed Ruvheneko Programme as a success story given that 80% of pregnant mothers volunteered to get tested and it was the first rural based institution to implement PMTCT.

### 1.2. Situating Ruvheneko Programme within the confines of social capital theory

To comprehend the modalities instituted in the fight against HIV and AIDS by the Ruvheneko Programme, the paper deploys the social capital theory as an analytical framework. As a conceptual tool, the notion of social capital provides a basis for explaining rational or purposive action within the constraints and opportunities of the social environment (Bhandari & Yasunobu, 2009). It refers to relationships and networks within specific localities, and can be used to explain relationships that extend beyond or between communities (Emmett, 2000). Another perspective on social capital proffered by Putnam (1993, p. 167) explains social capital as those ‘features of social organisation, such as trust, norms, and networks that can improve the efficiency of society by facilitating coordinated actions.’ Fundamental to social capital is that it facilitates cooperative behaviour as opposed to self-seeking and opportunistic behaviour (Bhandari & Yasunobu, 2009; Putnam, 1993). From a societal collective dimension, social capital as a resource benefits the entire community by facilitating community problem solving (Bhandari & Yasunobu, 2009; Coleman, 1990). Social capital has thus found applications in initiatives that strengthen what Hawe and Shiell (2000, p. 879) refer to as ‘natural helping networks’ for building empowerment and community capacity and develop contextual interventions.

In the light of the above, the impact of HIV and AIDS in Zimbabwe has warranted grassroots and community response at micro-level rather than wait for governments to intervene. Already, communities in rural Zimbabwe had their own problem-solving mechanisms in societies before the outbreak of HIV and AIDS through the existence of *Zunde raMambo* (chief’s granary). This helped in providing food to less privileged families with food from the common granary which all village members worked on. This mindset is supported by Putnam (1995) who asserts that citizen participation in community groups fosters social capital which brings about interpersonal trust, norms and networks which can influence health behaviour directly as a result of normative pressure to influence individual characteristics. Evans (1996, p. 167) affirms that, ‘if people cannot trust each other or work together, then improving the mental conditions of life is an uphill battle.’

This paper however is well aware of various criticism levelled against the social capital theory notably by Haines (2008), Navarro (2002), Fine (2001), and Mosse (2006). These scholars criticise the social capital theory for the general call for participation without accounting for the role of power, politics and economic forces determining participation and forming of associations. Participation is treated holistically as if everything happens in a natural setting. As will be demonstrated in the course of the discussion, issues of power and politics manifested in the running of Ruvheneko, though they are not accounted for by the social capital theory.

## 2. Aim of the study

In light of the above, this study sought to unpack the rural initiatives behind the establishment of the Ruvheneko Programme. Specific focus is on trying to understand how Ruvheneko Programme managed to thrive given its location within a resource constrained rural setting. In addition, the fact that Ruvheneko Programme has attracted so much media attention and described locally, nationally and internationally as a model of a sustainable and successful HIV and AIDS project in resource constrained rural settings (CDC, 2003; The Herald, 2003), creates the curiosity to find out more about the lessons that can be learnt from this programme. The following research questions therefore informed the main objective of the study:

1. What initiatives were put in place in establishing the Ruvheneko Programme?
2. How did Ruvheneko initiatives contribute to the fight against HIV and AIDS within a rural set up?
3. What mechanisms were instituted in forging partnership between the community and other stakeholders?

## 3. Methodology

The study was carried out using qualitative research methods and the approach was considered most appropriate in order to understand the contextual influences and the modalities that were taken in the establishment of the Ruvheneko programme. A qualitative approach was deemed useful in exploring complex issues such as people’s beliefs, perceptions and opinions concerning the issue under study (Babbie, 2007; Hennink, Hutter, & Bailey, 2011). A case study approach involving field research was utilised as the essential ‘building block’ of empirical research (Pierce, 2008). This allowed for ‘an empirical inquiry’ that investigates a contemporary phenomenon within its real life context’ where more than one source of evidence are explored to gather evidence from a given area (Yin, 2003, p. 13). In essence, a case study approach was also appropriate given the unique and interesting story about the rural initiative in mitigating the impact of HIV and AIDS in Chirumhanzu.

In the context of HIV and AIDS related studies, the inductive and descriptive nature of qualitative research provides in-depth understanding of the socio-behavioural aspects of the epidemic (Power, 1998). Qualitative methods enabled the researchers to critically reflect on, analyse and interpret different dimensions that surround the integration of the stakeholders in the Ruvheneko Programme.

### 3.1. Study site and research sample

The study was conducted at St Theresa’s Mission Hospital in the Midlands Province of Zimbabwe where Ruvheneko is housed. Chirumhanzu is a rural area which is occupied by predominantly Shona-speaking people. The discovery of gold in the 1990s (in Chinyuni, Mashamba and Mvuma areas) has seen gold-panning becoming one of the major activities attracting unemployed people from the surrounding rural communities. Many unemployed people in Chirumanzi have been trapped in gold-panning as an economic activity given that the area is overcrowded, rocky and dry, rendering it agriculturally unproductive. At the time that Ruvheneko was established, the discovery of gold saw many people flocking to the area for gold-mining. The life-style of the illegal gold-miners, popularly known in Zimbabwe as *Makorokoza* is usually associated with recklessness leading to risk sexual behaviour and the spread of sexually transmitted diseases and HIV and AIDS. Charandura growth point, which services St Theresa’s Hospital and other local government institutions, is also a centre where most of the *Makorokoza* come to enjoy their fortunes made from gold. In the process, this has attracted sex workers around the area, exacerbating the spread of HIV and AIDS.

St Theresa Hospital is one of the oldest health care Mission centres in Zimbabwe and is renowned for servicing resource constrained rural areas. The research sample consisted of 5 St Theresa’s Hospital Staff, 10 church members (5 women from St Anna’s women prayer group known as *Nzanga yaMbuya* Anna in the local parlance and 5 men from St Joseph’s men prayer group also known locally as *Nzanga yaJosefa*), traditional leadership (1 village headman from the nearby Mahaso village and 2 elders from the same village). This sample was chosen for practical reasons, especially to ensure easy management of both the participants and the collection of in-depth data. In general, 18 interviews were conducted with the objective to get a detailed and rich narrative on the phenomenon being investigated. The men and women from the prayer groups were selected from various villages served by Ruvheneko Programme. Choosing participants from diverse village settings helped to improve on the trustworthiness of the research findings as opposed to choosing participants from one locality. All the participants were purposively selected on the basis of their knowledge about the establishment of Ruvheneko, with the exception of Mahaso village which was chosen on the basis of accessibility and proximity to the hospital.

### 3.2. Data gathering approach

Guided by McMillan and Schumacher (2006), primary data was sourced from open-ended interviews and focus group discussions with the purposefully selected participants. In this case, participants were those believed to be knowledgeable about the establishment and operations of Ruvheneko. An interview guide was used to provide some structure during the interview process. Interviews, as noted by Creswell (2007) and Seamark and Lings (2004), were found appropriate because they allowed participants to explain their lived experiences and knowledge about the Ruvheneko Programme. On the other hand, 1 focus group discussion was conducted with 10 members who were randomly selected as follows: 2 from community caregivers, 2 from church women, 2 OVC, 2 from hospital staff, 1 from the youth and 1 village elder.

Utilisation of dual methods involving in-depth interviews and focus group discussion helped to ensure that specific information required for the purpose of the study was collected while at the same time leaving room for further probing. A combination of focus group discussions and interviews helped in ensuring data trustworthiness since the outcomes could be compared against each other. One of the strengths of interviews and focus group discussions experienced during the field research was that they allowed the informants enough time to think through their responses and to seek clarification when necessary (Babbie, 2007). Since the people of Chirumhanzu are dominantly Shona-speaking, Shona language was used as a medium of communication during the field interviews and interviewing people in their own language allowed them to express themselves freely. Fundamentally, a dual method approach involving focus group and interviews was deemed relevant in covering thematic aspects of the study such as the logic behind the establishment of the Ruvheneko Programme, aspects of community mobilisation, the role of the church and the community, stakeholder engagement, and how the entire project was sustained both socially and economically.

To minimise bias and possible researcher-interviewee tensions, the principal author, who was the interviewer for this study, avoided using leading questions. Guided by the importance of reflexivity when conducting field research (Yin, 2013), the principal author, who was neither known to the participants nor identified as part of Ruvheneko or one of the stakeholders, embraced her outsider identity to minimise bias and pre-determined judgements throughout the data collection process.

Apart from the collection of primary data, the study also benefitted from copious amount of secondary data sources in the form of official reports of the Ruvheneko Programme namely End of Phase 1 Report: May 2003–March 2006 and Ruvheneko Programme Report on Key Programme Achievements for the period (2003–2009). Reviewed also were St Theresa’s Hospital Annual Reports of 1999, 2001, 2009 as well as the St. Theresa’s Hospital PMTCT of 2009. Lastly, related literature on HIV and AIDS trends in Zimbabwe as well as official UNAIDS reports formed part of secondary data sources.

### 3.3. Data analysis

The first step on data analysis was the transcription of the interview outcomes from Shona to English language. Data were then analysed qualitatively using a thematic approach. This was accomplished by linking the emerging themes to the data collected (Thomas & Harden, 2008). These themes captured findings derived from research questions and objectives. Data was coded without ‘trying to fit it into a pre-existing coding frame’ (Braun & Clarke, 2006) or the researcher’s preconceptions or theoretical interests which would make the approach analyst driven and deductive in nature (Hayes, 1997). The analysis was therefore data driven and all the themes which were derived were solely from data findings, whether latent or explicit. All the themes, to be discussed later, emerged from data analysed thematically.

### 3.4. Ethical underpinnings of the study

Since this study involved human subjects, ethical clearance was secured from the Hospital authorities before the commencement of the study. In addition, the researchers ensured that ethical principles pertaining to voluntary participation, informed consent, confidentiality and anonymity were taken into consideration. Before the research proceedings commenced, the participants were all informed about the purpose of the study and that their participation was voluntary. They were also informed that should they decide to disengage from participating in the study, they were free to do so. All the informants were in their right frame of mind and were above the legal recommended age in Zimbabwe which is 18 years. To ensure confidentiality and anonymity, all informants were assured that information collected from participants was at all times going to be kept confidential and used only for the purposes of academic research. The right of the informants to provide information on the basis of anonymity during focus group discussions, interviews, field visits and life histories was observed. In fact, all the names in this study are pseudonyms (not real names) except for Dr. Stoughton only, which is a real name because his name appears in the secondary sources of data.

## 4. Findings

### 4.1. Chirumhanzu home based-care project

Findings from the interviews with the hospital staff revealed that what is now Ruvheneko Programme should be understood in the context of the pioneering project, the Chirumhanzu Home Based-Care Project which started in 1994. Mrs Moyana (pseudonym) none of the senior hospital official narrated that, ‘the project was merely an initiative by hospital health personnel, Dominican Sisters and expatriate Doctors who tried to address the challenge of increasing HIV and AIDS patients in the Hospital.’ The idea was also to help family members cope with the challenge of HIV and AIDS while working with volunteer Care-Givers who are supervised by professional nurses. The Home Based-Care approach adopted a comprehensive framework which included providing socio-psycho support to the infected and affected.

As presented in the St Theresa’s Hospital Annual Report (2001), ‘family is the most important resource for HIV and AIDS patients and that home care meets the needs of patients effectively.’ It also emerged that the Chirumhanzu Home Based Care Project derived insights from the 1993 World Health Organisation AIDS Home Care Handbook. One hospital official also reported that the project managed to survive financially because of support from the Swiss NGO Solidarmed. Another finding is that ‘the strategic partnership between traditional leadership and hospital personnel was meant to gain people’s trust, help people overcome issues of stigma and ignorance associated with HIV and AIDS.’ From the interviews, it was however revealed that this could not be achieved since there was no adequate involvement of local chiefs and communities, some even demanded payment. All these challenges revealed the fact that a more nuanced initiative was needed, in order to address the challenge of HIV and AIDS in Chirumhanzu; and other similar contexts in Zimbabwe and the continent at large.

### 4.2. Community mobilisation

The interviews with most of the hospital staff revealed that ‘community mobilisation was a key factor in mobilising the people of Chirumhanzu towards adopting a unified stance in the fight against HIV and AIDS.’ Mobilisation was therefore achieved through CHBC visits to several rural villages and households. The understanding at this point was that forming an effective partnership between the ‘users’ and ‘providers’ was the only path towards grassroots responses to the devastating impact of HIV and AIDS in the community. It emerged that the CHBC project was a precursor to the establishment of Ruvheneko in the sense that it marked the first hospital initiative where Care Givers could visit the sick in the comfort of their homes. The idea was to reduce stigma through teaching people on how to cope with the HIV and AIDS problem as well as supporting the infected psycho-socially, spiritually, medically, physically, and materially. As pointed out by Cephas (pseudonym) one of the Ruvheneko Programme senior officials, ‘CHBC was meant for capacity building in order to strengthen local community responses and understanding of the HIV and AIDS epidemic as well as encouraging participation.’ Community mobilisation was accomplished through various HIV and AIDS awareness campaigns in the form of poems, drama, and several visits to different communities serviced by the hospital. The activities carried out during the households and village visits included, teaching people about the impact of HIV and AIDS on individuals, friends and family; behaviour change; traditional practices and cultural norms which fuel the spread of HIV and AIDS; treatment of orphans and vulnerable children; as well as safer sex negotiation skills. It was reported that these mobilisation initiatives did not only attract community participation, but helped to break the silence about HIV and AIDS which indeed was not an easy topic to talk about openly because of the associated stigma and discrimination. Community mobilisation heralded a collaborative effort in the fight against stigma and discrimination that had exacerbated the raging virus. From the interviews with the hospital staff, it emerged that engaging the community through their leaders (who are the custodians of the people) helped to establish report and communication link between health professionals and the end users who are in this case the community.

### 4.3. Utilisation of religious ethos

In addition to community mobilisation, another finding is that focus group discussions and interviews revealed how women and men’s prayer groups utilised biblical ethos in persuading encouraging fellow community members to participate in HIV and AIDS projects/affairs. The initiative taken by the women’s prayer group in taking care of the sick, according to *Mai Mukuru* (St Anne’s sect group leader), is a religious obligation, and, to phrase it in her own words, … *kutenda kusina mabasa kwakafa mwanang*u. This translated in English means that faith without actions is dead my child. The point being conveyed in this context is that, acts of charity are considered as good deeds and a reflection of true faith, and it forms part of Christian ethos to do humanitarian responsibilities. This also conveys the message that Christians have a moral obligation to show compassion, love and integrity both individually and communally, and this is what the Ruvheneko VCGs and primary caregivers strive to achieve. In line with religious ethos, it also emerged that one key strategy that galvanised the initiative to establish Ruvheneko is the formation of the Chirumhazu Church Leaders Association (CCLA) in 2003. The CCLA is an interdenominational arrangement of various church leaders in the area meant to find solutions to the HIV and AIDS problem. According to Sekuru Murehwa (pseudonym) from the men’s prayer group, *Nzanga YaJosefa,*

… *utachiwana hwe HIV/AIDS hauzivi mutsauko wemasvondo uye kana vanhu vachidanana, kashoma kufunga nezvesangano. Izvi ndizvo zvinoreva kuti panoda kubatana kwedzisvondo mukurwisa chirwere ichi* … (the HIV virus knows no church boundaries and when people engage in sexual intercourse they rarely think about religious denominations and this is why we decided inter-denominations engagement with on the HIV/AIDS problem).

Through these religious ethos, other churches came to appreciate the need for proactive engagement in HIV and AIDS matters and it helped to break the perceptions about the HIV and AIDS epidemic as well as condom usage which most churches believed was responsible for promoting promiscuity.

### 4.4. Utilisation of cultural ethos on volunteerism

Complementing religious initiatives was also the aspect of volunteerism embedded in the Shona cultural and beliefs practices according to the Mahaso village headman *(sabhuku).* The headman gave an example of the idiom which fosters cooperation among the Shona people which says … *chaona hama hachisekanwi* (do not laugh when your neighbour is in trouble). In the traditional parlance, the idiom was and is used to discourage the community from spiting or laughing at those who are poor or experiencing problems. The idiom was later adopted in order to warn people against laughing and discriminating against People Living with HIV and AIDS (PLWHA). According to the Mahaso village headman, … *dama nderokuti seka urema wafa* … (the philosophy is that if one laughs, in future it might be them or their relatives suffering from HIV and AIDS and would need community support). In the light of these rich cultural precepts, helping others is thus seen as some kind of social investment since there is hope that community members will reciprocate the good deeds should one need the same help in future. These cultural and religious belief systems infused a sense of community belonging which encouraged many to participate in the establishment of the Ruvheneko Programme.

From the interviews with the two other village elders, Baba Garapo and Baba Muchihwa (pseudonyms), the formation of Ruvheneko also benefited from the Shona idiom … *gunwe rimwe haritswanyi inda* … which when directly translated means that one finger cannot kill lice. What emerged from the interviews with the village elders is that fighting HIV and AIDS cannot be separated from other traditional practices that discourage individualism in addressing social problems. It can therefore be argued that a blend of religious, social and cultural values became the foundation upon which community engagement and participation was fostered. In retrospect, the Ruvheneko programme can thus be construed as one of those home-grown solutions to rural community problems which had developed self-sustainable approaches to HIV and AIDS.

### 4.5. Stakeholder meeting initiative

Findings from the interviews and focus group discussions with various participants acknowledged the role of the April 2003 stakeholder meeting involving the Ministry of Health and Child Welfare (MoHCW); CCLA representatives; chiefs and headmen; the Ministry of Education (MoE), represented by school headmasters; and PACT Zimbabwe, representing the funders – Canadian International Development Agency (CIDA) and Swedish International Development Agency (SIDA). A principal from the local St Josephs’ school, Mr Mhara (pseudonym) narrated that ‘through the guidance of hospital leadership, the stakeholders at the meeting agreed to transform the CHBC from a project to a programme.’ This also involved choosing a new name for the CHBC and several names emerged from the consultations with the community members such as *Ruponeso* (Bringing back life), *Rusununguko* (Freedom)*, Ruvheneko* (meaning light) as it is understood in the Shona language. Amongst the suggested names, Ruvheneko was found to be the most appealing. Etymologically, Ruvheneko in Shona language designates ‘light’, ‘giving light’ or ‘brightness’.

According to *Mai Mukuru,* Mbuya Hwande (pseudonym) the name Ruvheneko helped to address derogatory labels such as ‘*Vanhu veAIDS*’, which means AIDS people, which were attached to the HIV and AIDS patients and those involved in HIV and AIDS projects. Such derogatory labels caused stigma, and most of the village care givers involved in the CHBC who were not infected were not comfortable with the name *Vanhu veAIDS.* More importantly, such derogatory labels are common talks at beerhalls, community gatherings and usually emerge from carefree people who do not sympathise or accommodate those infected or affected by HIV and AIDS. It can therefore be argued that the name Ruvheneko helped to address these concerns as it promised hope and a sense of collectivity among various stakeholders. Both the infected and affected could now work well without any negative connotation as expressed by one of the community leaders, according to the interview with Mr Gore Following the naming of the programme, stakeholders made it clear that the main objective of Ruvheneko is to reduce stigma surrounding HIV and AIDS by giving quality care to patients and to provide psycho-social support to the infected and affected.

### 4.6. A gendered approach to the fight against HIV and AIDS

An interesting finding from the establishment of the Ruvheneko Programme is the gendered dimension. This is reflected in the positive participation of men in the activities leading to establishment of Ruvheneko. In an interview with Mai Gono (pseudonym), one of the Hospital staff, it emerged that

the decision to involve men in HIV and AIDS matters stemmed from the realisation that HIV and AIDS knows no gender barriers and that leaving men out was going to complicate things since most of them were not going to allow their wives to participate in HIV and AIDS programmes. Moreover, most men in rural areas are involved in risk sexual behaviours and they end up infecting their wives and we realised that involving them brings a positive change.

In essence, Ruvheneko Programme brought a paradigm shift to the African traditional practices of relegating household chores like fetching water and caring for the sick to women and girl children only. This observation is in line with Familusi’s (2012) analysis of Yoruba culture, which like many other African cultures is castigated for relegating women to the background merely because of the patriarchal nature of society.

## 5. Discussion of findings

The aim of the study was to examine the initiatives that were put in place in establishing the Ruvheneko Programme and this was discussed in the context of the fact that Ruvheneko has been cited in newspapers and academic writings as a model of response to the human security threats posed by HIV and AIDS in resource constrained rural settings in Zimbabwe (CDC, 2003). Ruvheneko is renowned for being the first rural initiative in Zimbabwe to open the first HIV testing centre as well as instituting the PMTCT of HIV in pregnant women. The establishment of the Ruvheneko Programme demonstrates an interesting dimension of community engagement in the fight against HIV and AIDS as the tripartite partnership consisting of the Mission Hospital staff, the Church, and the community (including schools, villages, local chiefs, a village headmen) joined hands in mitigating the impact of HIV and AIDS in the area. Collaborative efforts of these various stakeholders in implementing the Ruvheneko programme demonstrates a holistic approach to fighting HIV and AIDS.

An interesting finding regarding all the initiatives towards the establishment of Ruvheneko is that the severity of HIV and AIDS in Chirumhanzu created some kind of fear of the unknown to the extent that members felt obliged to be part of the programme. This resonates with Rodriguez-Garcia’s (2013) model of social change that looks at how social challenges foster social cohesion and collectively problem solving approaches. This also reveals grassroots response to the impact of HIV and AIDS since the community improvised some self-help mechanisms in order to respond to such social challenges like AIDS (Rodriguez-Garcia, 2013). As noted by Schwobel (2006), it is precisely when human dignity is compromised that it becomes imperative for the church to engage in critical and affirmative public theology.

There is also an interesting dimension regarding the role of the Church in the founding of Ruvheneko Programme. Literature shows that the church in Zimbabwe was slow to respond to the impact of HIV and AIDS and this was premised on the belief that the epidemic was punishment from God (Clifford, 2004). Furthermore, the use of condoms was discouraged since it was said to be against religious ethos and that condoms were influencing the children to indulge in early sexual activities instead of abstaining from sex. In essence, the Roman Catholic Church is prided for being accommodating to some traditional African practices, and this cemented cooperation between the various churches and community members.

In addition to the role of the church, findings point to the important role of religious ethos in the establishment of Ruvheneko. There is a religious meaning ascribed to the name wherein the former St Theresa’s Hospital and patron for Ruvheneko, Dr. Stoughton, linked the name Ruvheneko to the biblical context in John 8 verse 1 2 which reads: ‘I am the light of the world’ (Mission Doctors Association Report, Heal the Sick, 2003). The idea of linking the light to HIV/AIDS was meant to give people hope and courage in the fight against HIV and AIDS. Literature also supports that the idea of Ruvheneko was invoked against the backdrop of trying to meet the needs of the infected and affected in the comfort of their homes and families so that the light and warmth brought to them by caregivers brings hope and eradicate the stigma associated with HIV/AIDS (Campbell, 2009). All these biblical traits in the founding of Ruvheneko illustrate the synergy between the community and the church in the battle against HIV and AIDS. An important point to note is that the idea of giving psycho-social support to the infected and affected as spelt in the mission statement of Ruvheneko is in line with ethical guidelines of the Zimbabwe National HIV/AIDS Policy of 1999; the Zimbabwe National Community Home-Based Care Standards of 2000; as well as the Zimbabwe National Home-Based Care Policy of 2001.

The element of volunteerism which is also core to the establishment of Ruvheneko Programme resonates with African traditional belief systems. Community participation and volunteerism in Chirumhanzu is perceived as a duty enshrined in cultural and moral values of the community. It is regarded as a sign of *Ubuntu*, translated in Shona as *unhu* or *hunhu,* meaning a responsible human being (Kaseke & Dhemba 2007, p. 91). Studies also show that volunteerism appeals directly to Africans’ natural religiosity rather than their material self-interest (Mbiti, 1969). Volunteerism, as depicted in the Shona cultural ethos of *Ubuntu*, thus sees homes shunning the individualistic or inward-looking families, to ones based on communalism (Kaseke & Dhemba 2007).

Effective community mobilisation is one initiative which played a pivotal role in engaging the community and the whole notion of community engagement can be understood in the context of the discourse of ‘participatory’ approach to development’ (Chambers, 1994; Emmett, 2000; Swanepoel & De Beer, 2006). Chambers (1994) describes this as ‘bottom-up’ approach to development in which people must be empowered and take full responsibility for their own development. This also entails that the role of authorities or NGOs must be enabling and supportive, which was the case in the formation of Ruvheneko Programme. What is unique about Ruvheneko if compared to similar projects like Mutambara Mother and Child Survival Training and Development is the ability of continuity or sustainability in the absence of donor support. In the case of Mutambara, the establishment was premised on top-bottom approach and the use of expatriates without capacitating the United Methodist Hospital Personal on HIV and AIDS and project management. The missing link between Ruvheneko and Mutambara case studies is that, in the case of Mutambara, they failed to understand that the role of development agents should be more of advisory than implementers themselves. This would allow sustainable implementation of the project in the absence the development agents.

It is therefore justifiable to assert that the establishment of Ruvheneko can be attributed to effective community engagement which allowed community participation and involvement. This manifested for example in the selection of the programme name after a wide consultation and debate with community members during the stakeholders meeting. The fact that the community members were actively involved in the selection of the programme name fostered a sense of ownership of the programme. In addition, experiences from other projects like Mashambanzou Care Trust partner organisations engaged communities through participation, they were not directly involved in the conceptualisation and planning of the projects. As such, community involvement was mainly limited to implementation and interventions like the OVC and HBC relying heavily on voluntary skills. Besides volunteer skills, community resource mobilisation has been marginal across the evaluated projects. Other similar projects like the one in Chimanimani show the crisis of involvement as a major setback to achieving success and sustainability in rural initiated HIV and AIDS programmes. In the case of Chimanimani, a study by Madzingira, Muhwava, and Mapfumo (2007) reveals that community members in Chimanimani complained that the Population Services Zimbabwe (PSZ) youth centre was being run like a private institution with little and insignificant input from the targeted population. Such reports point to the fact that instrumental participation is at play, adopting a top-bottom approach instead of the recommended bottom-up approach in contemporary developmental discourses.

Lastly, another finding regarding how Ruvheneko managed to appeal to the communities is that the idea of using poems and drama in raising awareness concurs with the notion of ‘edutainment’ raised by Goldstein, Usdin, Scheepers and Japhet (2005) in their discussion of the role of entertainment in HIV and AIDS education. They talk about how music and drama, for example, can be used as vehicles for both entertainment and educating people about HIV and AIDS, as in the case of Ruvheneko.

## 6. Conclusion and recommendations

The study has demonstrated that the severity of the human security threats posed by the scourge of HIV and AIDS led to the creation of an alliance between the church, hospital, and the community as a home-grown solution to the problem of HIV and AIDS in the Chirumhanzu rural area. It emerged that volunteerism which characterised community participation emanated from biblical, cultural and traditional ethos which contributed positively towards solving community problems, including HIV and AIDS. This article demonstrated that all the initiatives adopted in the establishment of the Ruvheneko Programme show the importance of a holistic approach in mitigating the impact of HIV and AIDS in resource constrained rural settings. The paper concludes that a holistic approach involving effective community mobilisation, religious and cultural ethos as well as the gendered approach, played a pivotal role in making Ruvheneko a unique rural initiative in responding to the human security threats posed by HIV and AIDS.
